# Low-Intensity Focused Ultrasound Targeted Microbubble Destruction Enhanced Paclitaxel Sensitivity by Decreasing Autophagy in Paclitaxel-Resistant Ovarian Cancer

**DOI:** 10.3389/fonc.2022.823956

**Published:** 2022-04-29

**Authors:** Gonglin Fan, Jiale Qin, Xiaofeng Fu, Xing Si, Liqiang Li, Keji Yang, Beibei Wang, Haiya Lou, Jiang Zhu

**Affiliations:** ^1^Department of Ultrasound, Sir Run Shaw Hospital, Zhejiang University School of Medicine, Hangzhou, China; ^2^Department of Ultrasound, Women’s Hospital, Zhejiang University School of Medicine, Hangzhou, China; ^3^State Key Laboratory of Fluid Power and Mechatronic Systems, School of Mechanical Engineering, Zhejiang University, Hangzhou, China; ^4^Center of Cryo-Electron Microscope (CCEM), Zhejiang University, Hangzhou, China

**Keywords:** ovarian cancer, paclitaxel, resistance, autophagy, low-intensity focused ultrasound, microbubble

## Abstract

Ultrasound targeted microbubble destruction (UTMD) was introduced as a promising method to improve anti-tumor therapeutic efficacy, while minimizing side effects to healthy tissues. Nevertheless, the acoustical phenomenon behind the UTMD as well as the exact mechanisms of autophagy action involved in the increased anti-cancer response are still not fully understood. Therefore, we examined the drug resistance-reversing effects of low-intensity focused ultrasound with microbubble (LIFU+MB) in paclitaxel (PTX)-resistant ovarian cancer cells. Cell viability was evaluated using CCK8 (Cell Counting Kit-8), apoptosis was detected by flow cytometry, quantitative real-time PCR and Western blot were used to detect the expressions of mRNA and protein, and autophagy was observed by transmission electron microscopy (TEM). We revealed that the level of autophagy was increased (*p <* 0.05) in PTX-resistant ovarian cancer cells. Treatment of LIFU+MB combined with PTX can notably inhibit proliferation as well as increase apoptosis (*p <* 0.01) in drug-resistant cells. We proposed that LIFU+MB might affect the sensitivity of ovarian cancer cells to PTX by modulating autophagy. To verify the hypothesis, we analyzed the autophagy level of drug-resistant cells after the treatment of LIFU+MB and found that autophagy was significantly inhibited. Altogether, our findings demonstrated that LIFU+MB could reverse PTX resistance in ovarian cancer *via* inhibiting autophagy, which provides a novel strategy to improve chemosensitivity in ovarian cancer.

## Introduction

Drug resistance is still a severe problem in the management of ovarian cancer all over the world (1). The standard postsurgical chemotherapy is the use of paclitaxel (PTX), a natural antimitotic agent, which has proven effective in many ovarian epithelial carcinomas (2). However, the treatment is often compromised by a high rate of relapse because of the development of drug resistance, which is a major obstacle in the management of ovarian cancer (3). Drug resistance has been linked to many mechanisms, including efflux transporters, apoptosis dysregulation, autophagy, cancer stem cells, epigenetics, and the epithelial–mesenchymal transition. Thus, it is key to developing and choosing effective therapies (4).

In recent years, therapeutic ultrasound, especially low-intensity focused ultrasound (LIFU), has received increasing attention due to its non-invasive nature, safety, and low cost (5). Ultrasound can cause physical and biochemical effects to affect tumor cell damage and apoptosis (6). A large number of recent studies have verified that ultrasound-targeted microbubble destruction (UTMD) technology can be applied for tumor-targeted therapy, providing a new treatment method for malignant tumors (7). Many UTMD-induced bioeffects have been reported already. For example, studies have shown that sonoporation can induce cellular stress, progression delays, endocytosis, actin cytoskeleton disruption, and membrane blebbing (8). However, the role of UTMD-induced autophagic changes in PTX-resistant cells remains unclear; thus, further studies are needed to elucidate its role.

Autophagy is an evolutionarily conserved indispensable catabolic process for the degradation of cytoplasmic components within lysosomes, which leads to organelle turnover and provides energy and macromolecular precursors (9). Autophagy frequently occurs during tumor growth and cancer chemotherapy (10). However, increasing evidence implicated that constructive autophagy usually functions to protect cancer cells during chemotherapy, leading to cancer drug resistance and refractory cancer (11). Recently, some studies have revealed that, under certain conditions, inhibiting autophagy can suppress cancer resistance (12).

Although the efficiency of UTMD-mediated cavitation in permeabilizing the biological barriers has been demonstrated, the exact mechanism behind the action of UTMD-induced autophagic changes has not been completely elucidated (13). Furthermore, the effect of UTMD in autophagy has not been tested yet. The main objective of this study was to explore the underlying mechanisms of clinically approved MB, in combination with LIFU in cancer therapy. Our data revealed that LIFU+MB was effective in cell apoptosis. The biological effects of LIFU+MB were significant determinants for the inhibition of cell proliferation, while we proposed that downregulated autophagy might be a contributing factor for improving chemosensitivity.

## Materials and Methods

### Cell Culture

PTX-sensitive ovarian cell lines SKOV3 and A2780, and PTX-resistant cell lines SKOV3-TR and A2780-TR were gifts from Women’s Reproductive Health Research Laboratory of Zhejiang Province (Hangzhou, China). A2780 and A2780-TR were cultured in RPMI 1640 media (Gibco, China) containing 10% fetal bovine serum (FBS, GIBCO or CellMax) and 1% penicillin/streptomycin (HyClone Laboratories, USA; 100 units/ml penicillin and 100 μg/ml streptomycin). SKOV3 and SKOV3-TR cells were cultured in McCoy’s 5A medium (Boster Biological Technology Co., Wuhan, China) supplemented with 10% FBS and 1% penicillin/streptomycin. To maintain the drug-resistant phenotype, SKOV3-TR and A2780-TR cells were cultured in the presence of 10 nmol/L PTX (Bristol-Myers Squibb Pharmaceuticals Ltd., USA) and passaged for 1 week in a drug-free medium before the experiment. All cells were grown in an incubator with a humidified atmospheric air containing 5% CO_2_ at 37°C. The cells were used for experiments on the logarithmic phase.

### Determination of the Inhibitory Concentration 50%

To identify the situation of the PTX resistance, cells were treated with increasing dosages of PTX for 48 h, and cell viability was tested by the Cell Counting Kit-8 (CCK-8, Dojindo Laboratories, Kumamoto, Japan) according to the manufacturer’s instructions. The absorbance at 450 nm was measured by using a Versamax microplate reader (Thermo Varioskan Flash, USA). Absorption in the blank well was subtracted, and the control was set as 100%, and others were normalized accordingly. The IC_50_ was calculated by the GraphPad Prism program.

### LIFU+MB Therapy System

**Figure 1:**
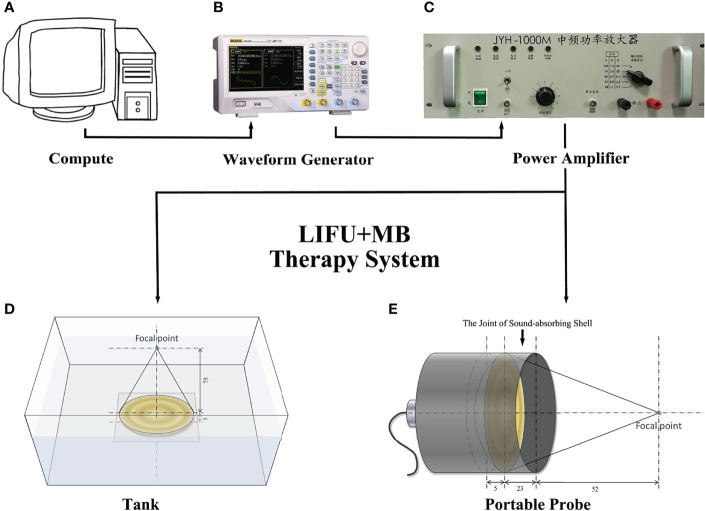
Design of acoustic exposure platform. Illustration of the entire platform setup that includes **(A)** compute, **(B)** waveform generator, **(C)** power amplifier, **(E)** transducer, and **(D)** the exposure platform immersed within a degassed water bath.

**Figure 2:**
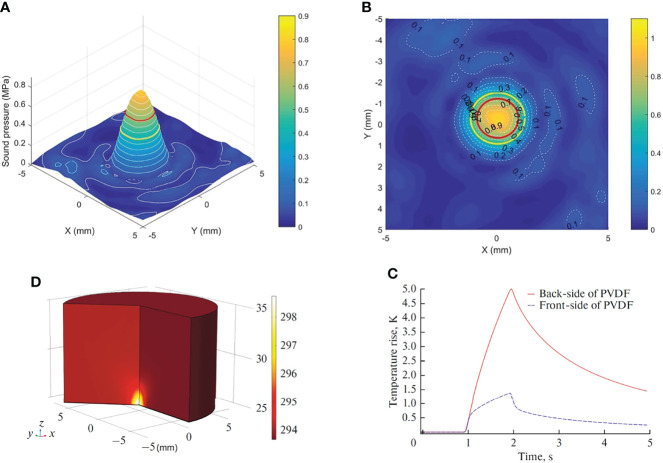
Characterization of the *in situ* ultrasound field and temperature change. **(A)** 3-D plot of the peak negative pressure on the hydrophone scan plane. **(B)** 2-D plot of the peak negative pressure. Red circle: −3 dB focal region. Yellow circle: −6 dB focal region. **(C)** Temperature rise in the sensor. **(D)** Temperature change at the front side and back side of PVDF.

The ultrasound probe system was developed and produced by our group (14, 15). We followed the best community-accepted practices in the tutorial papers as guidance (16, 17). The water tank type and the portable type are made using *in vitro* and *in vivo* experiments (**Figures 1A–E**). The system can deliver focused ultrasound energy (transducer’s active diameter 67 mm, −6 dB focal diameter 2.44 mm, −6 dB focal length 5.0 mm). In *in vitro* experiments, acoustic absorbing layers were placed at the inner wall of the tank, consisting of a computer, a waveform generator (RIGOL DG1022U, Beijing RIGOL Technology Co., Ltd., China), a power amplifier (JYH-1000, JIE HUI Industry Co., Ltd., Shanghai, China), and a motion stage to allow for movement and positioning perpendicular to the ultrasound beam axis. The animal experiment was performed using a portable ultrasonic probe (the cover of the probe is made of a sound-absorbing material), which was also designed and produced by our group. The beam profiles were recorded using a needle hydrophone (HNR-0500; Onda, Sunnyvale, CA, USA) and an automated three-axis micro-positioner (ASTS-01; Onda Corporation). **Figures 2A, B** show the corresponding peak negative pressure on the hydrophone scan plane. **Figures 2C, D** show the simulation results of the temperature rise in the PVDF sensor when the ultrasound transducer worked for 1 s (15).

The SonoVue^®^ microbubble contrast agent (Bracco S.p.A, Milan, Italy) was reconstituted in 5 ml of normal saline, a concentration of 10% (v/v) of which was used in each experimental group. Once reconstituted, it can be chemically and physically stable for 6 h. Studies showed that SonoVue^®^ microbubbles are safe and well tolerated when administered intravenously at 0.03 and 0.3 ml/kg, which are the expected clinical dose and 10 times the clinical dose, respectively. The extremely rapid pulmonary elimination of the compound would indicate that SF_6_ does not accumulate in healthy subjects, even with repeated administration. SonoVue^®^ microbubbles can also serve as artificial and preexisting nuclei for ultrasound-induced stable and inertial cavitation, which are considered to be the most important mechanism for therapeutic applications. The mean terminal half-life was 12 min (range, 2 to 33 min). More than 80% of the administered sulfur hexafluoride was recovered in exhaled air within 2 min after injection and almost 100% after 15 min (18).

**Figure 3:**
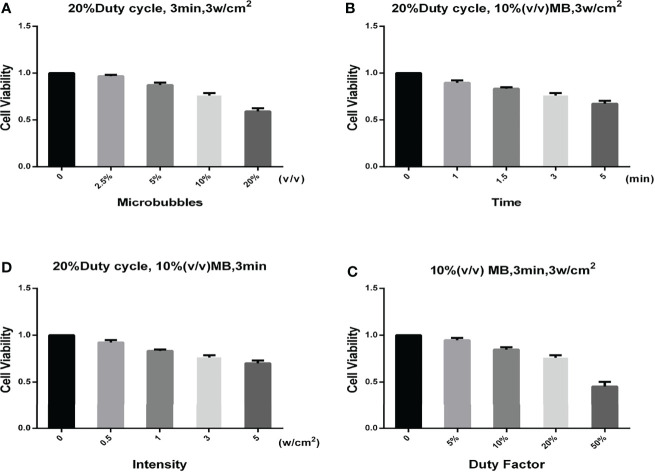
Relationship between different parameters and the cell viability. **(A)** Relationship between the SonoVue^®^ microbubble concentration and the cell viability. The frequency, duty factor, treatment time, and ultrasonic intensity were 1 MHz, 20%, 3 min, and 3 W/cm^2^, respectively. **(B)** Relationship between the treatment time and the cell viability. The frequency, duty factor, SonoVue^®^ microbubble concentration, and ultrasonic intensity were 1 MHz, 20%, 10% (v/v), and 3 W/cm^2^, respectively. **(C)** Relationship between the ultrasonic intensity and the cell viability. The frequency, duty factor, SonoVue^®^ microbubble concentration, and treatment time were 1 MHz, 20%, 10% (v/v), and 3 min, respectively. **(D)** Relationship between the duty factor and the cell viability. The frequency, SonoVue^®^ microbubble concentration, treatment time, and ultrasonic intensity were 1 MHz, 10% (v/v), 3 min, and 3 W/cm^2^, respectively.

The ultrasonic stimulation experiments were conducted in cells with and without microbubbles. **Figure 3** shows the relationship between SKOV3 cell viability and the concentration of SonoVue^®^ microbubbles, treatment time, ultrasonic intensity, and duty factor. During the experiments, the system operating parameters were 1 MHz, 20% duty cycle (on 0.2 s, off 0.8 s), 3 min exposure duration, 0.42 MPa peak negative pressure, 3 W/cm^2^ *I_SPPA_*, 0.6 W/cm^2^ *I_SPTA_*, and 108.0 J/cm^2^ with delivered acoustic energy density. The ultrasound beam propagates vertically into the target through an acoustically transparent sealed chamber (NUNC™ OptiCell^®^, cat. no. 155330; OptiCell^®^ is a unique cell culture format for growing, monitoring, and transporting cells, which consists of two parallel gas-permeable and cell culture-treated polystyrene membranes), with acoustic coupling provided by degassed water.

### Cell Proliferation

The *in vitro* experiment included eight groups: (1) Blank, (2) LIFU alone, (3) MB alone, (4) LIFU+MB, (5) PTX (200 nM), (6) PTX (200 nM)+LIFU, (7) PTX (200 nM)+MB, and (8) PTX (200 nM)+LIFU+MB. For the Blank and LIFU alone groups, an equivalent volume of medium was used instead of MB or PTX. After 0 h, 12 h, 24 h, 48 h, and 72 h, the medium was exchanged and cell viability was determined as mentioned above. All assays were replicated three times.

### Cell Apoptosis Analysis

Apoptosis of the cells was measured by an Annexin V-fluorescein isothiocyanate (FITC)/propidium iodide (PI) apoptosis detection kit (MultiSciences Biotechnology, Zhejiang, China) according to the manufacturer’s instruction. The cells were randomly divided into the aforementioned eight treatment groups. In brief, cells were harvested after 24 h and double-stained with Annexin V-FITC and PI for 15 min in the dark and then analyzed using a FACSCalibur flow cytometer (BD LSRFortessa, USA). The experiments were replicated at least three times.

### Western Blot Analysis

Cultured cells were harvested and lysed in RIPA buffer (Beyotime Biotechnology, Shanghai, China) containing 1% protease inhibitor and separated by electrophoresis, transferred to membranes, and subjected to Western blot according to the standard procedure. The following antibodies were used: Anti-LC3B (ab192890, 1:1,000) was purchased from Abcam (Cambridge, MA, USA), anti-SQSTM1/p62 (#8025, 1:1,000) was purchased from Cell Signaling Technology (Danvers, MA, USA), and anti-GAPDH (AF0006, 1:1,000), used as the internal control, was purchased from Beyotime Biotechnology (Beyotime Biotechnology, Shanghai, China). After incubation with the appropriate secondary antibody, results were detected using ECL detection reagents. Immunoreactive bands were quantified by densitometry using ImageJ software.

### Transmission Electron Microscopy

The cells were collected after centrifugation at 1,000*g* for 5 min. Subsequently, the cells were washed twice with cold phosphate-buffered saline (PBS) and fixed with 2.5% glutaraldehyde overnight at 4°C. Then, cells were dehydrated in a graded ethanol series and embedded by epoxy resin. After mounting on copper grids, the ultrathin sections were double-stained with uranyl acetate and lead citrate. The samples were examined and imaged with a TECNAI 10 or 12 transmission electron microscope (Philips, Holland).

### RNA Preparation and Quantitative Real-Time PCR

Total RNA was extracted using TRIzol reagent (Invitrogen, USA), and RT-PCR was performed according to the manufacturer’s instructions. cDNA was synthesized using the PrimeScript RT reagent Kit (Code No. RR047A, Takara, Dalian, China). RT-PCR was performed using the Roche LightCycler 480 and TB Green™ Fast qPCR Mix (Code No. RR820A, Takara, Dalian, China). The primer sequences are listed in **Table 1**. All reactions were run in triplicate. The expression level was normalized to the GAPDH control, and relative expression values were determined against the control using the 2^–ΔΔCT^ method.

**Table 1:** Sequences of primers for quantitative real-time PCR.

Gene Name	Forward Primer Sequence (5′→3′)	Reverse Primer Sequence (5′→3′)
GAPDH	GGAGCGAGATCCCTCCAAAAT	GGCTGTTGTCATACTTCTCATGG
p62(SQSTM1)	AAGCCGGGTGGGAATGTTG	CCTGAACAGTTATCCGACTCCAT
LC3B-II	GATGTCCGACTTATTCGAGAGC	TTGAGCTGTAAGCGCCTTCTA

### Animal Experiment

Animal experiments were approved by the medical experimental animal care commission of Zhejiang University. Female nude mice, aged 4–6 weeks, weighing 18–20 g, were purchased from the Animal Experiment Center (Shanghai SLRC Experimental Animal Co., Ltd., China) and were maintained under controlled conditions of temperature (20 ± 1°C, relative humidity 50%–80%) and illumination (12-h light, 12-h dark). All mice had free access to a standard diet and water.

Approximately 1×10^7^ A2780-TR cells in 200 μl of serum-free medium and Matrigel solution were injected directly into the flank of each mouse. The tumor-induced nude mice were randomly divided into four groups, with four mice in each of the following groups: the control group, the PTX (10 mg/kg)-treated group, the LIFU+MB-treated group, and the PTX (10 mg/kg)+LIFU+MB-treated group. When the tumor volume reached 150 mm^3^, the mice received a corresponding treatment. The therapeutic transducer was positioned on top of the tumor. An acoustic absorbing pad was placed under the animal to prevent standing waves from developing. PTX injection is a sterile, stabilized solution of PTX, suitable for dilution for intravenous administration. All the mice were anesthetized with intraperitoneal injections of 1% sodium pentobarbital (6.25 ml/kg), and different suspensions (0.2 ml) were administered *via* the lateral tail vein catheterized with a 26-gauge angio-catheter (Angiocath, Becton Dickinson, UT). For ultrasound stimulation experiments, the animals were exposed to tumor-directed acoustic insonation immediately after injection. The acoustic setup and insonation parameters are described as before. The mice were treated, the long and short diameters of the tumor were measured, and the tumor volumes (TV, TV = 0.5 × length × width^2^) were calculated every 3 days. The tumor growth curve was drawn according to the measured TV values. After 18 days of treatment, the nude mice were sacrificed by cervical dislocation, and the tumor mass was removed and weighed.

### Contrast-Enhanced Ultrasound Imaging Technique

In a preliminary study, contrast-enhanced ultrasound (CEUS) was performed in mice. The examinations were performed using a GE LOGIQ 9 unit (GE Healthcare, Waukesha, USA) and a linear probe (9L), adjusted to examinations using a microbubble contrast agent. SonoVue^®^ was used at a dose of 0.1 ml, immediately followed by an injection of 0.1 ml of normal saline. Images were recorded for 3 min after contrast agent injection.

### Histological Analysis and Immunohistochemistry

Tumor tissues from the control and LIFU+MB groups were embedded in paraffin and cut into 4 μm-thick sections. Then, the sections were stained with hematoxylin and eosin (H&E). For immunohistochemical staining, the sections were incubated with Anti-LC3B antibodies at 4°C overnight. The subsequent sections were exposed to HRP-antibody colored with diaminobenzidine (DAB), and visualized by microscopy (DM 2500, Leica, Germany).

### Statistical Analysis

All statistical analyses were performed using SPSS 19.0 software (SPSS Inc., Chicago, IL, USA). Data were presented as mean ± standard deviation. One-way analysis of variance (ANOVA) was used to analyze the differences among groups. The Student’s *t*-test and Mann–Whitney *U*-test were used to compare the means of the two continuous data. All contingency tables and multiple parameters were assessed using the chi-squared test. *p-values < 0.05, **p < 0.01, or ***p < 0.001 (two-tailed) were considered as statistically significant. Images were processed using GraphPad Prism 6 (GraphPad Software, La Jolla, CA, USA) and Adobe Photoshop CS5 (Adobe, San Jose, CA, USA).

## Results

### The Sensitivity of PTX in Human Ovarian Cancer Cell Lines SKOV3, A2780, SKOV3-TR, and A2780-TR

**Figure 4:**
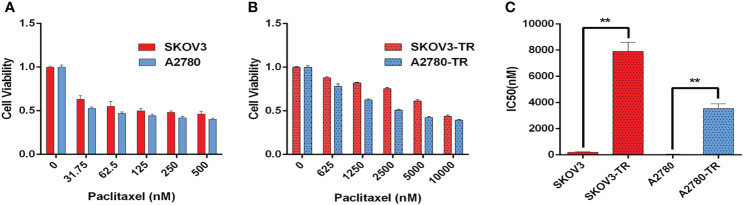
Paclitaxel inhibits ovarian cancer cell viability. **(A, B)** Viability of paclitaxel-resistant (SKOV3-TR and A2780-TR) and paclitaxel-sensitive (SKOV3 and A2780) cell lines in response to different concentrations of paclitaxel measured by the CCK-8 assay. **(C)** IC_50_ values of paclitaxel for SKOV3, A2780, SKOV3-TR, and A2780-TR cells. The IC_50_ values are calculated by SPSS 19.0 software. (**p < 0.01, n = 3, student t-test, means ± 95% CI).

SKOV3, SKOV3-TR, A2780, and A2780-TR cells were treated with increasing concentrations of PTX for 48 h (**Figures 4A, B**). Then, CCK-8 assay was used to detect the cells’ viability. Cell survival was decreased in a dose-dependent manner in all cell lines. As expected, the SKOV3-TR and A2780-TR cells were more resistant under different concentrations of PTX, as compared with the SKOV3 and A2780 parental cells (*p <* 0.05). IC_50_ values confirmed these results (**Figure 4C**). The IC_50_ values of PTX for SKOV3 vs. SKOV3-TR and A2780 vs. A2780-TR cells were 206.6 ± 28.9 vs. 7,914.1 ± 675.5 and 47.4 ± 3.9 vs. 3,538.0 ± 363.9 nM, respectively.

### The Increasing Autophagy Status in Cell Lines SKOV3-TR and A2780-TR

**Figure 5:**
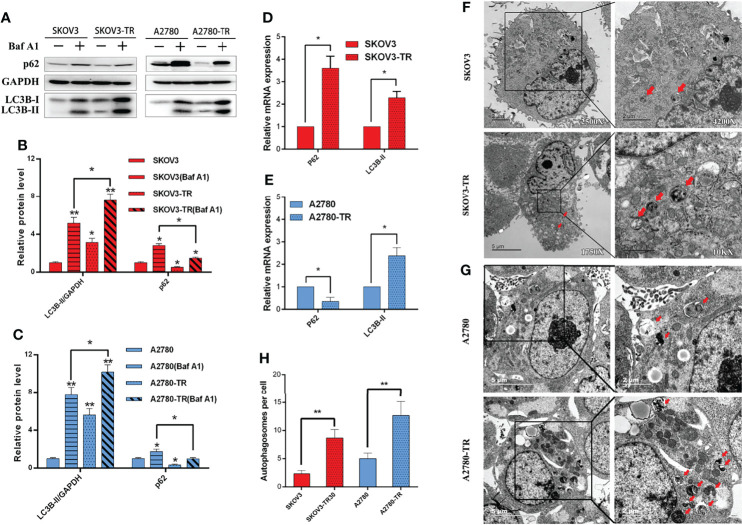
Autophagy detection in SKOV3, A2780, SKOV3-TR, and A2780-TR cells. **(A–C)** Western blot showed p62(SQSTM1) and LC3B levels in SKOV3, A2780, SKOV3-TR, and A2780-TR cells. The densitometric evaluations are calculated by ImageJ. **(D, E)** The expression of autophagy was determined by real-time PCR. **(F–H)** The autophagic vacuoles (autophagosomes) were detected in SKOV3, SKOV3-TR, A2780, and A2780-TR by transmission electron microscopy (TEM). The representative TEM images are shown and the typical autophagosomes are marked with red arrows. The number of autophagosomes per cell was calculated by counting the number of double-membrane organelles in 10 cells. (*p < 0.05, **p < 0.01, n = 3, student t-test, means ± 95% CI).

To determine the contributions of autophagy to cell chemosensitivity, the autophagy level was evaluated in SKOV3, SKOV3-TR, A2780, and A2780-TR cells. Compared with the PTX-sensitive cells, PTX-resistant cells showed higher LC3B-II protein level in Western blot analysis. For the change of p62/SQSTM1, it was inconsistent in two cell lines, which increased in A2780 but declined in SKOV3 (**Figures 5A–C**). Furthermore, autophagic flux was measured by assessing LC3B-II and p62/SQSTM1 in the presence of BafA1, an inhibitor that blocks the lysosomal degradation of autophagosomes. Consistently, PTX-resistant cells had higher levels of autophagic flux compared with the sensitive cells (**Figures 5A–C**). The RT-PCR results showed that the LC3B-II expression level was decreased, while the p62/SQSTM1 expression level was increased in the PTX-sensitive cell lines compared with the PTX-resistant cell lines. These results indicated that autophagy activity was higher in PTX-resistant cells than that in PTX-sensitive cells (**Figures 5D, E**). As expected, transmission electron microscopy (TEM) revealed the increasing number of autophagosomes in the SKOV3-TR and A2780-TR cells compared to the SKOV3 and A2780 cells (**Figures 5F–H**). Therefore, these results suggested that the higher level of autophagy in SKOV3-TR and A2780-TR cells might associate with the drug resistance character.

### LIFU+MB Recovered the Chemosensitivity of SKOV3-TR and A2780-TR Cells to PTX

**Figure 6:**
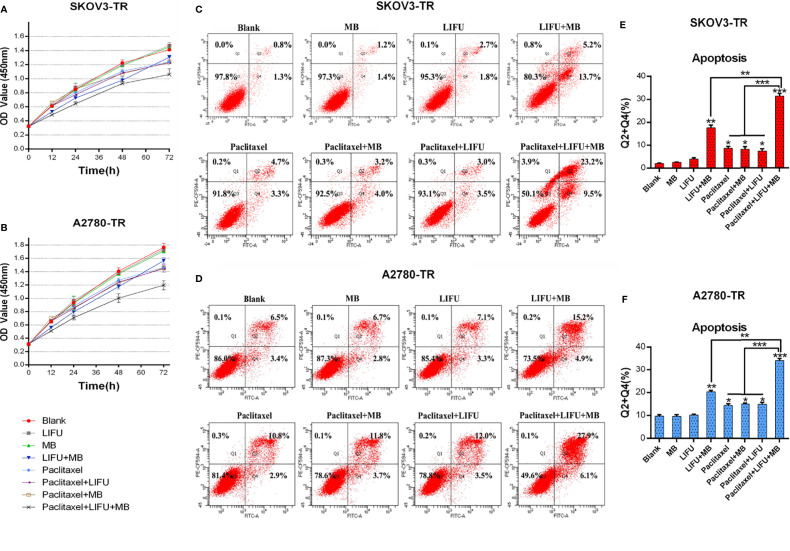
LIFU+MB enhanced the chemosensitivity of SKOV3-TR and A2780-TR cells to paclitaxel. **(A, B)** OD values were assessed by the CCK-8 method in response to different treatment for different times. **(C–F)** Analysis of apoptosis rates by flow cytometry and quantified after different treatment. (*p < 0.05, **p < 0.01, ***p < 0.001, n = 3, student t-test, means ± 95% CI).

Cell viability assays demonstrated that the treatment of LIFU and MB alone did not influence cell growth, but LIFU+MB inhibited cell proliferation to some extent. PTX+LIFU and PTX+MB treatments were unable to enhance chemosensitivity, while the PTX+LIFU+MB combination can distinctly inhibit cell growth of SKOV3-TR and A2780-TR cells (**Figures 6A, B**). In addition, the enhancement effect of LIFU+MB on the cells is short term.

To determine whether LIFU+MB treatment exerted a pro-apoptotic effect on cells, flow cytometry analysis *via* Annexin V-FITC/PI staining was performed. The results demonstrated that apoptotic cell death was induced upon LIFU+MB treatment while US and MB treatment did not. Consistent with these results, PTX+LIFU+MB significantly induced apoptosis compared with PTX treatment (**Figures 6C–F**). These results suggested that LIFU+MB treatment was able to enhance drug-induced apoptosis.

### LIFU+MB Decreased Autophagy Status of SKOV3-TR and A2780-TR Cells

**Figure 7:**
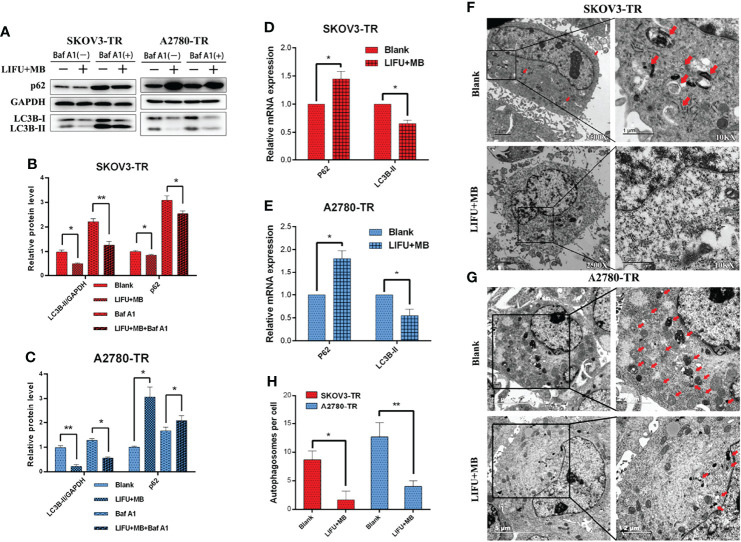
Autophagy detection in SKOV3-TR and A2780-TR cells after LIFU+MB. **(A–C)** Western blot showed p62(SQSTM1) and LC3B levels in SKOV3-TR and A2780-TR cells. **(D, E)** The expression of autophagy was determined by qRT-PCR. **(F–H)** Analysis of autophagy after LIFU+MB in SKOV3-TR and A2780-TR cells. (*p < 0.05, **p < 0.01, n = 3, student t-test, means ± 95% CI).

To further figure out the major factors of LIFU+MB treatment-induced chemosensitivity, we tested the autophagic rate after treatment. SKOV3-TR and A2780-TR cells were pretreated with autophagy inhibitor Baf A1 for 1 h, treated with LIFU+MB, and cultured for 24 h; the autophagic rate was detected later. Western blot analyses demonstrated that LC3B-II expression levels were decreased in the treatment group, and p62/SQSTM1 expression level was increased in A2780-TR but decreased in SKOV3-TR (**Figures 7A–C**). In the presence of Baf A1, the LIFU+MB group had lower levels of autophagy compared with the control. These observations were independently validated using TEM. LIFU+MB treatment strikingly decreased the number of autophagosomes in both SKOV3-TR and A2780-TR cells with or without BafA1 (**Figures 7F–H**). In addition, mRNA levels were also detected by qRT-PCR, and the data showed that LC3B-II expression level was decreased, while the p62/SQSTM1 expression level was increased after treatment (**Figures 7D, E**). These results suggested that downregulated autophagy of LIFU+MB treatment could contribute to chemosensitivity.

### LIFU+MB Improved PTX Efficiency in Ovarian Cancer *In Vivo*

**Figure 8:**
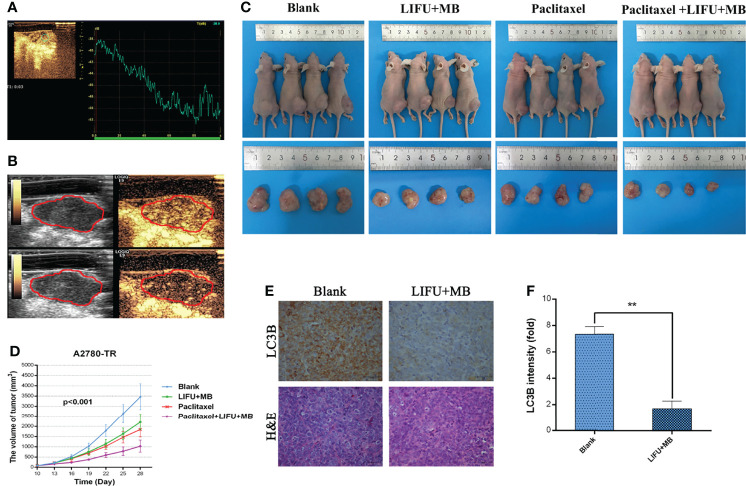
The effects of different treatments on the tumor-induced nude mice. **(A)** Contrast-enhanced ultrasound with time–intensity curve. **(B)** Preliminary assessment of tumors in mice, images at 15 s and 2 min after administration of 0.1 ml microbubble. **(C)** LIFU+MB significantly inhibited subcutaneous tumor growth in mice treated with paclitaxel. **(D)** Growth curve of A2780-TR subcutaneous xenograft tumors. **(E, F)** LC3B expression analyzed by immunohistochemistry in tumor tissues and quantified. Scale bar: 50 μm (×400). (**p < 0.01, n = 3, student t-test, means ± 95% CI).

The tumors showed complete hyperenhancement during the arterial phase in a preliminary experiment (**Figure 8A**). During the late phase, the tumors were also hypo-enhanced (**Figure 8B**). Next, the effects of LIFU+MB treatment on PTX efficiency were tested *in vivo*. The results showed that PTX and LIFU+MB treatment slightly suppressed tumor growth, whereas the LIFU+MB and PTX combination significantly enhanced the effect of PTX, suppressing tumor growth (**Figures 8C, D**). Immunohistochemical analyses showed that LIFU+MB decreased LC3B expression levels (**Figures 8E, F**). Our findings indicated that LIFU+MB treatment could sensitize ovarian cancer cells with PTX resistance *in vivo*.

## Discussion

Drug resistance is a major problem in chemotherapy for ovarian cancer (19). Numerous studies have found that autophagy dysregulation might play important roles in chemoresistance (20). Autophagy has dual roles in cancer. It may allow cells to survive under unfavorable conditions. Paradoxically, it may also emerge as a tumor suppressor and eventually kill cancer cells (21). In this study, we observed that the autophagy levels of PTX-resistant cell lines SKOV3-TR and A2780-TR were higher than their parental cells, SKOV3 and A2780 (*p <* 0.05), indicating that autophagy may play a pro-survival role here, consistent with the report from Zhang et al. (22). As a further layer of complexity, upregulation of autophagy may lead cancer cells to a non-proliferative dormant state that protects the cells from toxic injuries while preserving their stem-like properties (23).

In this study, we designed and constructed a LIFU therapeutic system, aiming to explore the efficacy and mechanisms responsible for the enhancement of combining UTMD+PTX treatment for refractory ovarian cancer. Early studies showed that UTMD could increase the permeability of the plasma membrane, and the mechanical and cavitation effects were considered responsible for the low-intensity ultrasound bio-effects (6). Cavitation divides into two different physical processes, stable and inertial, that affect cells in different ways (24). Stable cavitation refers to the periodic expansion and contraction of microbubbles around their equilibrium radius in a low-pressure sound field. Inertial cavitation refers to the large expansion and rapid collapse of microbubbles in a high-pressure sound field, and may trigger sonochemical reactions, generate reactive oxygen species, and cause sonoluminescence (25, 26). However, in our experiment, only LIFU (1 MHz, 3 W/cm^2^, 20% duty cycle) had little influence for cells, indicating that the inertial cavitation (LIFU+MB) may be a key factor.

To further explore the association between LIFU+MB and the level of autophagy, apoptosis and autophagy were evaluated in this study. Our results showed the increasing apoptosis rate of PTX-resistant cells, but the expression of LC3B-II decreased after treatment of LIFU+MB (*p <* 0.05). These results suggested that the LIFU+MB-induced cell death is achieved by inducing apoptosis. Indeed, therapies such as chemotherapy can induce stress, and the metabolic contributions from autophagy can help ameliorate the detrimental cellular effects (27). High-density stress conditions such as LIFU+MB may disrupt these metabolic compensations and induce apoptosis (28). Others, such as LIFU+MB-induced stress response, resembles cellular responses to electroporation and pore-forming toxins in membrane repair, thus restoring cellular homeostasis, and may lead to cell death (29). We also noticed that the effect of LIFU+MB did not last long *in vitro*. Thus, to enhance the effect of LIFU+MB treatment, the tumors were treated every 3 days *in vivo*.

During autophagy, the adaptor protein p62/SQSTM1 is consumed, and LC3B conversion is promoted. LC3B has two forms in cells: LC3B-I and LC3B-II, which is the central protein in the autophagy pathway. LC3B-I residing in the cytosol is converted to the membrane-bound LC3B-II during autophagosome formation (30). Thus, initiative autophagic flux can be indicated by LC3B-II amount and the accomplishment of p62/SQSTM1 degradation status, respectively (31). In this study, we found that LC3B-II expression level decreased after LIFU+MB treatment, suggesting that LIFU+MB could inhibit LC3B-mediated autophagy in PTX-resistant ovarian cancer cells. Nevertheless, further work needs to be performed to detect autophagy with the LIFU+MB and PTX combination.

Paradoxically, we found that basal p62/SQSTM1 expression was higher in SKOV3-TR cell lines, which is fundamentally different from what we found in A2780-TR cells. Following treatment with LIFU+MB, the level of p62/SQSTM1 decreased in SKOV3-TR cells while it increased in A2780 cells. Interestingly, qPCR revealed that the level of p62/SQSTM1 increased in two cell lines. In a previous study, p62/SQSTM1 was related to the resistant mechanism of SKOV3 cells, not only PTX but also other drugs (32). A possible explanation was that ovarian cancer is a heterogeneous disease in which different histological types may result from different origins and distinct genetic patterns (33). Because p62 contains several interaction domains to many signaling molecules for their proteasomal degradation, monitoring p62 degradation thus cannot accurately evaluate the exact autophagic flux or autophagy outcome (34). Moreover, whether downregulating the expression of p62/SQSTM1 can effectively sensitize PTX-resistant tumor is also not known (32, 35).

*In vivo*, we believe that the therapeutic efficacy observed can be attributed to not only the potential decrease of autophagic level in the tumor, but also other pathways. It is known that ultrasound in combination with microbubbles can increase the fenestration size of tumor's vascular wall, allowing deeper drug penetration. (36). It can also disrupt the tumor microenvironment and can affect many aspects of tumor biology such as hypoxia, vascular permeability, and interstitial fluid pressure (37). Nevertheless, further work needs to be performed to ascertain the true mechanisms behind the improved therapeutic efficacy in tumor tissue (38).

However, our studies have some limitations. First, cavitation is typically evaluated on a cell monolayer, allowing direct contact between the target cell line and microbubbles *in vitro*. Second, further experiments are needed to better understand the upstream regulation of autophagy and the related pathways in ovarian cancer. Moreover, whether the method presented here is effective in other refractory tumors is not known and require additional work.

## Conclusions

We have studied the efficacy and potential mechanism of UTMD+PTX treatment on refractory ovarian carcinoma. Our study indicated that LIFU+MB could enhance the localized anticancer effect of PTX *via* inhibiting autophagy. Hence, reversal of drug resistance is significantly improved by low-intensity focused ultrasound with microbubbles. In a more general sense, the combined LIFU+MB+chemotherapy drug treatment might offer an innovative way to effectively reduce the drug dosage so as to minimize the side effects of conventional chemotherapy.

## Data Availability Statement

The raw data supporting the conclusions of this article will be made available by the authors, without undue reservation.

## Ethics Statement

The animal study was reviewed and approved by the medical experimental animal care commission of Zhejiang University.

## Author Contributions

JZ and JQ designed research, revised the manuscript, and revised the final version of the manuscript. GF performed the experiments and drafted the article. LL, KY, XF, XS, and BW helped perform the research, contributed new reagents/analytic tools, and analyzed data. All authors contributed to the article and approved the submitted version.

## Funding

This work was supported by the National Natural Science Foundation of China (grant numbers: 81974470 to JZ, and 81601515 and 82171939 to JQ) and the Natural Science Foundation of Zhejiang Province of China (LY18H180001 to JZ and LY16H180003 to HL).

## Conflict of Interest

The authors declare that the research was conducted in the absence of any commercial or financial relationships that could be construed as a potential conflict of interest.

## Publisher’s Note

All claims expressed in this article are solely those of the authors and do not necessarily represent those of their affiliated organizations, or those of the publisher, the editors and the reviewers. Any product that may be evaluated in this article, or claim that may be made by its manufacturer, is not guaranteed or endorsed by the publisher.
